# Prevention and control of COVID-19 by primary health care facilities in China: a field-survey-based qualitative study in three typical cities

**DOI:** 10.1186/s12913-022-07770-4

**Published:** 2022-03-26

**Authors:** Yun-yun Yan, Teng-yang Fan, Yan-ling Zheng, Hai-qin Yang, Tian-shu Li, Hai-tang Wang, Yan-feng Gu, Xue Xiao, Zhao-hui Du, Xiao-ming Sun

**Affiliations:** 1 Zhongshan Hospital, Shanghai Medical College of Fudan University, 180 Fenglin Road, Xuhui District, 200030 Shanghai, China; 2 Affiliated Hospital of Zunyi Medical University, 149 Dalian Road, Huichuan District, Guizhou Province 563000 Zunyi, China; 3 Wuchang District Shouyilu Street Community Healthcare Center, Wuhan, China; 4 Wuchang District Huanghelou Street Community Healthcare Center, Wuhan, China; 5 Yachuan Township Health Center, Fenggang County, Zunyi, Guizhou Province 564200 China; 6 Pudong New District Shanggang Community Healthcare Center, 360 Changli Road, Pudong New District, 200120 Shanghai, China

**Keywords:** COVID-19, Community-based, Prevention and control, PHC facilities

## Abstract

**Background:**

During the coronavirus disease 2019 (COVID-19) containment, primary health care (PHC) facilities inChina played an important role in providing both healthcare and public care services to community populations. The tasks of COVID-19 containment facilitated by PHC facilities were different among different regions and during different periods of COVID-19 pandemic. We sought to investigate the gaps on task participation, explore existing problems and provide corresponding solutions.

**Methods:**

Semi-structured face-to-face interviews with COVID-19 prevention and control management teams of PHC facilities were conducted. Purposive stratified sampling was used and 32 team members of 22 PHC facilities were selected from Wuhan (as high-risk city), Shanghai (as medium-risk city) and Zunyi (as low-risk city). Framework analysis was employed to analyze the transcribed recordings.

**Results:**

The main tasks of PHC facilities during the early period of the pandemic included assisting in contact tracing and epidemiological investigation, screening of populations at high-risk at travel centers/internals, house-by-house, or pre-examination/triage within PHC facilities; at-home/ centralized quarantine management; the work of fever sentinel clinics.

Further analyses revealed the existing problems and suggestions for improvement or resolutions. Regular medical supply reserves were recommended because of the medical supply shortage during the pre-outbreak period. Temporarily converted quarantine wards and centralized quarantine centers could be used to deal with pressures on patients’ treatment and management of the febrile patients. Only after strict evaluation of nucleic acid testing (NAT) results and housing conditions, decision on quarantine at-home or centralized quarantine centers could be made. Settings of fever sentinel clinics at PHC facilities allowed fever patients with no COVID-19 infection risks for treatment without being transferred to fever clinics of the designed secondary hospitals. Psychological intervention was sometimes in need and really helped in addressing individuals’ mental pressures.

**Conclusions:**

During the COVID-19 containment, PHC facilities in China were responsible for different tasks and several problems were encountered in the working process. Accordingly, specific and feasible suggestions were put forward for different problems. Our findings are highly beneficial for healthcare teams and governments in handling similar situations.

**Supplementary Information:**

The online version contains supplementary material available at 10.1186/s12913-022-07770-4.

## Background

As gatekeepers of healthcare system, primary care providers (PCPs) are crucial for the coronavirus disease 2019 (COVID-19) control and surveillance in the community [1]. In South Africa, PCPs were responsible for identifying COVID-19 patients and managing mild cases at-home [2]. Haldane V and her colleagues reviewed 17 documents from 14 countries’ national health authority with regard to primary care response to COVID-19 service delivery, they reported these guidelines made recommendations mainly for how to ensure continuous providing of essential primary care services via telehealth or other virtual care modalities [3] Similarly, telephone triage and consults on COVID-19 related problems were PCPs’ major tasks in Flanders [4]. In India, some of the most tasks primary health care (PHC) facilities engaged in including COVID-19 case finding and referral [5]. Clearly, although the ultimate goal is to successfully contain the prevalence of COVID-19, the specific strategies and tasks of PCPs varied in different areas all over the world.

In China, more than four million primary care providers from 970 thousand PHC facilities including community healthcare centers (CHCs), township hospitals, community health service station and village clinics [6] involved in community-based prevention and control of COVID-19 [7]. During COVID-19 pandemic, the preventive and controlling policies were enacted here in China on the principle of region-specific measures, and its cities/areas were classified as high-risk, medium-risk and low-risk areas. Of which high-risk area refers to COVID-19 patients significantly increased in the district and the incidence rate was over 3/100,000 within one week. And the low-risk area refers to no new patients were confirmed in the district during the last 14 days [8, 9].

Additionally, COVID-19 pandemic was summarized to have three periods, which referred to the pre-outbreak, outbreak period and regular prevention and control period. The pre-outbreak period referred to the period from the initial report of unexplained pneumonia cases to the strict travel restrictions in Wuhan, ranging from December 31,2019 to January 23,2020. The outbreak period ranging from January 23, 2020 to March 17, 2020 [10]. Correspondingly, the regular prevention and control period began after March 17, 2020, when no newly infected patients were identified in Hubei Province. Growing to better understand the disease and the changing trend of COVID-19 pandemic locally and globally, with their focus shifted accordingly [11].

On the other hand, the unevenly distributed medical resources in different intro-city locations could not be overlooked [12]. Larson PS et al. described that urban-rural designation was an important factor in predicting epidemiological risk of infectious disease [13]. Based on the factors mentioned above, PHC facilities at different risk levels, with different intro-city locations might engaged varied tasks during different COVID-19 pandemic periods in China.

We all know that Wuhan had been a high-risk city until 18th March, 2020. On 21th August, 2020, Zhuqiao Town of Pudong New District, Shanghai has been identified as medium risk areas [14]. And Zunyi, Guizhou Province has been at low-risk before this research was designed. Beside this, Shanghai is an international economically developed city in eastern China, Wuhan is a megacity in central China, and Zunyi is an economically underdeveloped city in western China.

Because of areas at different risk levels, at different intro-city locations and different periods of COVID-19 pandemic might affected the task participation of PCPs, PHC facilities from Wuhan, Shanghai and Zunyi were selected to investigate the task difference and the existing problems during COVID-19 prevention and control across China.

## Methods

### Study design

Semi-structured face-to-face interviews were conducted in COVID-19 prevention and control management teams at PHC facilities from Wuhan, Shanghai and Zunyi from 2nd November,2020 to 24th December, 2020. Each interview including two sections, a questionnaire-based question answering and an in-depth interview.

### Questionnaire and interview item development

Firstly, we performed comprehensive literature search and attended online experience sharing academic conferences to understand the profile of PHC facilities’ tasks during COVID-19 containment. Then, we interviewed five PCPs (two from Shanghai, one from Wuhan and two from Zunyi) from 4 PHC facilities via telephone to learn the specific tasks which were conducted at each facility against COVID-19 prevention control. After that we designed the first draft of questionnaire on task participation of PCPs during COVID-19 prevention and control in China. The questionnaire would be revised and used for subsequent national survey schedule.

The in-depth interview contains items as follows: (1) the tasks of their PHC facility with respect to COVID-19 prevention and control; (2) the differences in the prevention and control priorities and tasks during the pre-outbreak, outbreak, and regular prevention and control periods; and (3) the existing problems and suggestions for improvement. Both the in-depth interview and the questionnaire response was anonymous and only basic demographics of the participants covered age, sex, education, profession, technical title, and working years was collected. The interview lasted 30–60 min.

### Participants

#### PHC facilities

The participants were chosen using purposive stratified sampling as follows.

 The PHC facilities were selected: (1) from Wuhan, Shanghai and Zunyi respectively according to their representative risk levels; (2) in each city 1–2 PHC facilities chosen from its urban, urban-rural and rural areas. In this section, only community- or township-based PHC facilities were targeted, community health service station and village clinics were excluded because they were subordinate unit of CHCs or township hospitals.

#### COVID-19 prevention and control management teams at PHC facilities

This research aimed to explore the specific tasks conducted by PHC facilities during the whole COVID-19 prevention and control period. The inclusion criteria were as follows: (1) COVID-19 prevention and control management teams at PHC facilities; (2) participated in COVID-19 prevention and control tasks in-person; (3) engaged in COVID-19 containment tasks during the whole pandemic period. Those who refused to be interviewed were excluded.

At each PHC facility, one or several members of COVID-19 prevention and control management team in charge of COVID-19 prevention and control tasks were invited to the face-to-face interview. The number of interviewees depended on if different members were responsible for different tasks. For example, if three team members were assigned to different tasks, all of them would be among our interviews.

### Data collection and analysis

 Oral informed consent was obtained from all participants before the interview, and this procedure was approved by the Ethics Committee of Pudong Institute for Health Development, Shanghai. None of the interviewees refused to participate in the study. All the interviews were conducted separately at the office of the interviewees or at the conference room of each PHC facility.

One well-trained researcher conducted the audio-recorded interviews. Recordings were transcribed verbatim by another researcher after each interview was done. When both the two researchers considered no new themes emerged from the interviews, it was believed that theoretical saturation was achieved. Once theoretical saturation was reached, continuous sampling of each city ceased. The final theoretical saturation decision of this research would be made after discussion by all the team members of the project. Supplementary tele-interview would be employed as alternatives if needed.

The recorded data were fully transcribed and analyzed manually. When transcribed, the recordings were analyzed using framework analysis with five steps: data familiarization, indexing, theme collating, thematic framework summary, and interpretation. The indexing was conducted by two researchers independently using Microsoft Excel (version 2019). The next step was to sort the codes into potential themes or sub-themes. After that all research members were required to attend a meeting to solves the following problems: (1) if the data was adequate enough to cover the study topic; (2) comparing concepts and themes to data, discuss whether the themes fully reflected the information provided by interviewees; (3) whether the current data achieved theoretical saturation; and (4) and discussion of disagreements across researchers. The research group concluded that theoretical saturation was reached after 32 interviews and no supplementary interview was conducted. Based on the meeting the themes or sub-themes were confirmed and summary of thematic framework was established under the cooperation of two researchers. The second discussion across authors achieved an agreement in the final thematic framework which consisted of the following categories (Table 1).

**Table 1:** The summary of themes and sub-themes

Themes	Sub-themes
Task participation	During the pre-outbreak period (Table S2); During the outbreak period (Table S3); During the regular prevention and control period (Table S4);
Main tasks at their PHC facilities	Flowchart of main task fulfillment by PHC facilities during regular COVID-19 prevention and control period (Fig. 1)
The existing problems	A shortage of medical supplies (during the pre-outbreak period); Pressure on patients’ treatment and management of the febrile patients (city at-high risk during the outbreak period); At-home or centralized quarantine? Febrile patient management (city at low-risk); Transferring; Screening at travel centers/intervals (low-risk area); Mental pressures.
Suggestions for solutions or improvement	Supplies storage; Graded diagnoses and treatments; Temporarily converted quarantine wards and centralized quarantine centers (city at high-risk); Strict evaluation of housing environment or transferring to centralized quarantine centers; Fever sentinel clinics at PHC facilities; Transferring; Screening might could be performed by well-trained non-medical workers; Psychological intervention.

*PHC* Primary health care, *PCP* Primary care provider

### Basic information of the PHC facilities and interviewees

In the field surveys, we obtained the first-hand information of 22 PHC facilities (Table S1) from the three different cities, 7 in Shanghai, 6 in Zunyi and 9 in Wuhan (including 1 in Ezhou adjacent to Wuhan), and 32 members in management teams responsible for different tasks of COVID-19 prevention and control participated in the interview. Of 32 interviewees (Table 2), 15 came from the PHC facilities in Shanghai, 8 from Zunyi, and other 9 from Wuhan.

**Table 2:** Participant descriptive characteristics

Characteristics	No. of participants (%; *N *= 32)
**Age(years)** Mean ± SD	33–67(45.53 ± 6.54)
**Sex**	
Male	16(50)
Female	16(50)
**Education**	
Junior college	4(12.5)
College	24(75)
Graduate school	4(12.5)
**Profession**	
General medicine	13(40.63)
Clinical medicine	8(25)
Traditional Chinese medicine	2(6.25)
Public Health	2(6.25)
Management	1(3.13)
Nursing	5(15.63)
Stomatology	1(3.13)
**Technical title**	
Senior	3(9.38)
Associate senior	14(43.75)
Intermediate	12(37.5)
Junior	2(6.25)
Others^a^	1(3.13)
**Years of work experience** Mean ± SD	2 ~ 45(21.59 ± 9.08)

^a^ Others: administrative personnel with no medical technical title

#### Perspective 1: Task summary of the PHC facilities

##### Flowchart of the main tasks during regular COVID-19 prevention and control period

**Fig. 1:**
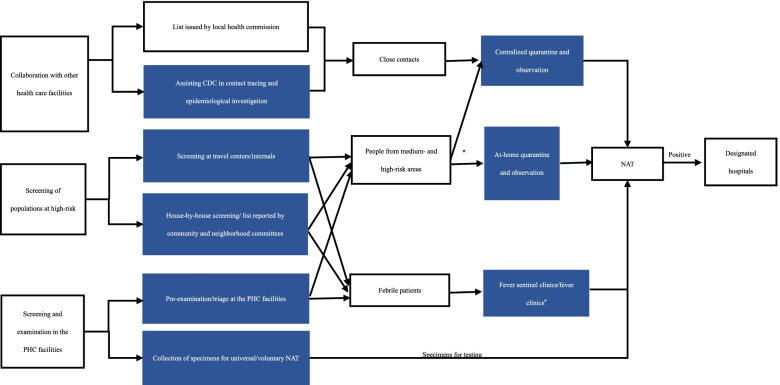
Flowchart of main task fulfillment by PHC facilities during regular COVID-19 prevention and control period. CDC=Center for Disease Control and Prevention, PHC =primary health care, NAT=nucleic acid testing. Nodes in blue as the main tasks in the PHC facilities. * Special populations refer to older people (age 70 years and above), minors (age 14 years and below), women who are pregnant or in traditional postpartum confinement, people with mobility difficulties, people with older people or children in need of care, and people with underlying diseases and are not suitable for centralized quarantine. Special populations should be diagnosed using nucleic acid testing at examination sites. Those with negative results and proper housing conditions can apply for at-home quarantine and health

As indicated in Fig. 1, the main tasks during regular COVID-19 prevention and control period at the PHC facilities were to assist the Center for Disease Control and Prevention (CDC) in contact tracing and epidemiological investigation, and to screen high-risk populations at the travel centers/intervals, at the community households, as well as within the PHC facilities. Those who had returned from medium- and high-risk areas and their close contacts were transferred to at-home/centralized quarantine, and those who had fever to a fever sentinel clinic or a fever clinic. NAT was performed on the high-risk populations and febrile patients, and those who were tested positive were transferred to the designated hospital for a standard treatment.

#### Perspective 2: The existing problems

##### A shortage of medical supplies (during the pre-outbreak period)

Undoubtedly, the understanding of COVID-19 and SARS-CoV-2 was limited during the pre-outbreak period. There was a severe shortage of medical supplies at the PHC facilities to mitigate the respiratory infectious disease of COVID-19. The shortage still remained in the form of N95 masks, surgical masks, protective suits, isolation gowns, goggles and protective screens, even though some governments, social organizations, individuals and international channels were active in raising and donating across the world. Consequently, some disposable materials had to be reused for the time being upon strict disinfection during the pre-outbreak period.

*We didn’t know what could help spread this infection, and we didn’t realize the early contagious severity before a wide transmission.* [PCP 22, female, from Wuhan]

*The protective supplies were really insufficient, so we had to purchase substitutes for temporary use, such as raincoats, shoe covers, self-made face screens and so on. The medical staff members in high-risk positions like the pre-examination triage personnel, had the priority of wearing protective suits during the period of shortage.* [PCP 16, female, from Wuhan]

*One protective suit was reused for seven times after disinfection when we screened febrile patients at the highway turnpike on January 23, 2020.* [PCP 21, female, from Wuhan]

##### Pressure on patients’ treatment and management of the febrile patients (city at-high risk during the outbreak period)

During the field investigation, 6 of 9 PHC facilities in Wuhan took the responsibility of treating the infected patients, as one of the main tasks during the outbreak period, which was significantly different than in other cities. There was a markedly increased urgent need of sick beds to provide proper and prompt treatment for COVID-19 patients. Since fever was a typical and initial symptom of COVID-19 infections, febrile patients were individuals at high-risk to be screened out for standardized management.

*We were the first medical team to support the fever clinic of the secondary hospital in our district. We had a large number of febrile patients gathering here in the clinic. The sick beds were severely limited for the infected patients.* [PCP 23, male, from Wuhan]

*We identified suspected patients with a fever and other respiratory symptoms as the NAT and CT were not accessible at the PHC facilities in the early time.* [PCP 22, male, from Wuhan]

##### At-home or centralized quarantine?

In quarantine, individuals at high-risk were managed under the centralized quarantine, and those who had a proper living place at home for quarantine could apply for domestic quarantine. Initially, at-home quarantine was recommended, but the problem was that cross-infection could not be strictly avoided as this respiratory infectious disease was transmitted rapidly through droplets or daily contacts, so that centralized quarantine became the main management style.

*At our PHC facility, we have received 59,500 persons, and 26,000 of whom were aliens, the Japanese accounting for 80%. We managed 80% individuals under at-home quarantine in Changning District of Shanghai. At-home quarantine places high demands on the living conditions. Centralized quarantine was recommended in Shanghai.* [PCP 05, male, from Shanghai]

##### Febrile patient management (city at low-risk)

In China, the rural areas, which have much to be desired in economics and infrastructure, tend to have limited access to medical resources. Accordingly, the rural citizens were at a relatively lower risk with infectious disease which could be transmitted by close contact with infected patients. When screened out, the febrile patients had to be transferred to the fever clinic of the designed secondary hospital.

*Febrile patients were all transferred to the fever clinic of the designed secondary hospital, even if they didn’t have an out-of-town trip, or had no contact with such a traveler, which otherwise could increase the financial burden and cross-infection risk.* [PCP 28, male, from Zunyi]

*The fever clinic of the county hospital can’t meet the medical needs of the citizens living in the rural areas. It’s usually far away. It’s difficult to be accessible. And the medical expenses could be high. So the problem was that, 90% of the fever patients could be excluded from the possibility of COVID-19 infection using epidemiological history inquiry.* [PCP 27, male, from Zunyi]

*The fever clinics have been set up since SARS epidemic in 2003. During the COVID-19 outbreak, as of November 17, 2020, around 600 people with a fever visited the fever clinic, and only 15 were transferred to the superior hospital for additional medical care.* [PCP 15, male, from Shanghai]

##### Transferring

As a growing number of individuals at high-risk needed to be transferred, ambulances were not enough; they were only used to meet the need of critically ill patients for transferring.

*All individuals at high-risk need to be transferred but the ambulances were not enough. Thus transferring has become a risk factor of cross infection.* [PCP 19, female, from Wuhan]

##### Screening at travel centers/intervals (low-risk area)

As a low-risk area, Zunyi had its tasks focus on screening and management of individuals at high-risk.

*In Zunyi, staffs from all the PHC facilities were arranged on duty around the clock, to screen high-risk populations such as febrile patients and arrivals from Hubei province at highway turnpikes during the outbreak period.* [PCP 25, male, from Zunyi]

##### Mental pressures

During the pandemic of COVID-19, infected patients, suspected patients, people under quarantine, medical workers as well as populations in general were very likely to suffer a mental pressure.

*Two critically ill COVID-19 patients died when transferred to the designed hospitals, even though they had received a proper and prompt treatment. This left on me a long-term impact. So I would say psychological intervention is important to both patients and medical workers.* [PCP 18, male, from Wuhan]

*The individuals who were under the centralized quarantine sites would have anxiety or feel lonely, which would cause their disobedience to the quarantine management. But they can understand this procedure with the help of patient explanations.* [PCP 26, female, from Zunyi]

#### Perspective 3: Suggestions for improvement

##### Supplies storage

Personal protective equipment (PPE) and other needed medical supply reserves were prepared at PHC facilities.

*But for now, the protective supplies reserves were prepared for at least one month’s utilization by all staffs. Other PHC facilities are the same.* [PCP 16, female, from Wuhan]

##### Graded diagnoses and treatments

At the PHC facilities, where graded diagnoses and treatments were performed on the patients with different symptoms, managed the confirmed patients with mild symptoms and suspected patients, and transferred the critically ill patients after diagnosis.

*One of the critical tasks for the PHC facilities was to identify the critically infected patients under the limited conditions before transferring them to the superior hospital timely.* [PCP 18, male, from Wuhan]

##### Temporarily converted quarantine wards and centralized quarantine centers (city at high-risk)

Quite a number of general wards were temporarily converted to quarantine wards to accommodate a growing demand for sick beds (Figure S1) and hotels or school dormitories were converted to centralized quarantine centers.

*We were the first medical team to support the fever clinic of the secondary hospital in our district. There were 13 medical workers from several PHC facilities, and 12 from the Red Cross in Jianghan District, who participated in repurposing the maternity hospital to be a designated hospital with 230 beds, one for one. All staff members here, medical or non-medical, including volunteers, didn’t get infected with SARS-CoV-2 during the working period of 3 months.* [PCP 23, male, from Wuhan]

*A separate management was applied to patients with mild symptoms, suspected patients with COVID-19, their close contacts, and febrile patients at the centralized quarantine sites after Feb 3, 2020.* [PCP 20, female, from Wuhan]

##### Strict evaluation of housing environment or transferring to centralized quarantine centers

At-home quarantine places a high demand on the living conditions, on the ground that the high population density in the city could increase the possibility of family cluster cases of COVID-19 infection (Figure S2). Therefore, centralized quarantine was recommended as the first choice in these areas (Figure S3).

*The evaluation of living conditions should be performed by a three-man panel including one local police, one neighborhood committee worker and a PCP. They were also responsible for the follow-up health monitoring.* [PCP 05, male, from Shanghai]

*With the increasing number of returnees from abroad, 10 centralized quarantine sites were set up to be managed by 13 PHC facilities of Jiading District of Shanghai.* [PCP 11, female, from Shanghai]

##### Fever sentinel clinics at PHC facilities

Because the treatment provided at the fever clinic within the designated hospital could increase the cost, the risk of cross infection and the waste of medical resources during the first half of 2020, pilot fever clinics were established at the PHC facilities in Wuhan, Beijing, and Shanghai, respectively.

*A sentinel clinic was set up at our PHC facility for febrile patients so that the medical service became accessible and convenient after March 2020.* [PCP 11, female, from Shanghai]

##### Transferring

In the high-risk city of Wuhan, the transferring from the communities was mainly done on the vehicles owned by the neighborhood committees.

*The grid managers were responsible for transferring using the designated vehicles, and the individuals were transferred to the 4 designed hospital in our district.* [PCP 19, female, from Wuhan]

##### Screening might could be performed by well-trained non-medical workers

Screening at travel centers/intervals has become a routine procedure during regular control and prevention period and this task was in charged by workers of travel centers/intervals instead of PCPs during the outbreak period (Figure S4).

*It is difficult for limited medical staffs to handle both the daily clinical works and COVID-19 prevention and control task*s. *I think that screening at travel centers/intervals might could be performed by their own workers at the first time. And suspected individuals at high-risk could be further evaluated by PCPs.* [PCP 28, female, from Zunyi]

##### Psychological intervention

Psychological consultation was essential for both publics and medical staffs.

*I remembered that one day I answered more than 200 telephone calls. Of course, most of them didn’t have any physical problems. They only wanted to share their anxiety with a medical staff.* [PCP 21, female, from *Ezhou*]

### Statement of principal findings

This research identified task participation, existing problems and suggestions for improvement about tasks of PHC facilities during COVID-19 prevention and control. The main tasks of PCPs in China including assisting in contact tracing and epidemiological investigation. Populations at high-risk were screened at travel centers/internals, house-by-house or via pre-examination/triage at the PHC facilities; and next they were kept in quarantine at-home or transferred to centralized quarantine and observation. Febrile patients were transferred to fever sentinel clinics/fever clinics for further examinations.

Additionally, several problems were pointed out by the interviewees in task implementation, and corresponding solutions or recommendations were developed respectively.

Firstly, during the pre-outbreak period, there was a shortage of medical supplies. Therefore, after the outbreak period, PHC facilities in mainland China were recommended to reserve medical supplies regularly in case of necessity. Moreover, healthcare system of Wuhan faced a big challenge of limited capacity in managing COVID-19 patients and individuals at high-risk. Quite a number of quarantine wards and centralized quarantine centers were temporarily converted to address this problem.

The team members also noted that cross-infection could not be strictly avoided in at-home quarantine as the strong infectivity of SARS-CoV-2. Growing to better understand the disease, only after strict evaluation of NAT results and housing conditions, decision on quarantine at-home or centralized quarantine centers could be made. On the other hand, settings of fever sentinel clinics at PHC facilities allowed fever patients with no COVID-19 infection risks for treatment without being transferred to fever clinics of the designed secondary hospitals. Besides, mental pressure was likely to be experienced among all publics so that psychological counseling was sometimes in need and really helpful.

### Strengths and limitations

This is a field-survey-based study of the tasks performed at PHC facilities in China during the COVID-19 pandemic, and face-to-face interviews were conducted with management teams from different intra-city geographic locations in three typical cities including Wuhan, Shanghai, and Zunyi. This research identified task participation, existing problems and suggestions for improvement about tasks of PHC facilities during COVID-19 containment. But there are several limitations in this research. Firstly, it’s a qualitative study and no quantitative data was collected. Secondly, this research was conducted during 2nd November,2020 to 24th December, 2020, and other significant task including COVID-19 vaccination fulfilled by PHC facilities in China was not mentioned because it was implemented later on. Additionally, this research did not follow strict random sampling and the sample size was small. But we followed purposive stratified sampling and used thematic framework to analyze the data. Questionnaire based national survey would be conducted next.

### Interpretation within the context of the wider literature

#### Emergency reactions to COVID-19 pandemic

Medical supply shortage was a worldwide challenge faced by medical systems at the initial stage of COVID-19 pandemic [15, 16] PHC facilities here in China have undergone similar situations. To prevent the occurring of similar conditions in future infectious disease control process, PHC facilities in mainland China were recommended to reserve PPEs and other medical supplies which could be used for at least one month by all staffs. Another problem should be noted is that healthcare system in Wuhan faced a challenge on limited capacity in managing all patients and individuals at high-risk identified during such a short period of time. In addition to the construction of shelter hospitals [17] and infectious disease hospitals [18], quite a number of general wards were temporarily converted to quarantine wards to accommodate a rapidly growing demand for sick beds. And hotels or school dormitories were converted to centralized quarantine centers to manage individuals at high-risk [19]. According to the data from official website of the National Health Commission of People’s Republic of China, no new COVID-19 patient was confirmed after March 18th, 2020 in Hubei Province because of the series of effective emergency reactions facilitated by the governments, and medical or non-medical staffs [20]. The working experiences were worth sharing, as reference for similar situations.

#### Accurate management with region-specific, class-specific, population-specific measures

The PHC facilities had unique advantages in accurate population management, for which they were not replaced by the secondary and tertiary hospitals. The refined and grid-based management was proved to be effective in epidemiological investigation, contact tracing, screening, transferring, quarantine and general population management. During the regular prevention and control period in China, the pandemic was characterized by cluster cases; therefore, the prevention and control policies were modified to redefine the minimal unit of risk areas. In the high-risk areas, the minimal units were schools, buildings, factories, workplaces, and natural villages. Intensive monitoring and disinfection were conducted to facilitate accurate management [21]. On the other hand, what can be learned from Wuhan is that graded diagnoses and treatments were performed on the patients with different symptoms.

#### Psychological intervention

We learned that mental pressure was likely to be experienced among COVID-19 patients, populations at high-risk, related workers like medical staffs, and even the public under COVID-19 pandemic conditions [22, 23]. Psychological problems resulting from quarantine should not be ignored. Therefore, the management of quarantined populations should include psychological interventions. At-home quarantine was likely to increase patient comfort and improve their well-being [24], whereas centralized quarantine could probably cause psychological problems, such as anxiety and mania [25]. Mental support from family members and friends can have a positive impact on the psychological status of quarantined individuals [26]. On the other hand, the psychological conditions of medical staffs should also be addressed, as required by PCP 18 from Wuhan.

#### At-home/centralized quarantine

Quarantine strategies, especially those implemented during the early period of outbreak, are important to controlling infectious diseases [27]. In the pre-outbreak period in China, the populations at high-risk were mostly monitored under at-home quarantine. This was validated by the interview with PCP 26 from Zunyi. However, at-home quarantine places a high demand on the living conditions, and the high population density in the urban areas could increase the possibility of family cluster cases of COVID-19 infection [28]. Therefore, the quarantine strategy was adjusted, based on the earlier experiences. A centralized quarantine strategy was adopted during later periods in Wuhan. As PCP 20 said, the divisions of febrile patients, patients suspected of COVID-19 infection, and close contacts were respectively made for observation, diagnosis and treatment at the centralized quarantine sites. In Shanghai, the centralized quarantine was preferred for those who had returned from the high-risk areas, and the at-home quarantine was only practiced on those who had an acceptable living place for strict monitoring. The populations at high-risk in Zunyi of Guizhou were also mainly managed to be receive centralized quarantine. As to COVID-19 prevention and control, therefore, we recommend the at-home or centralized quarantine strategy could be recommended by PCPs after strict evaluation. The quarantine management can be actualized by the PHC facilities, as manifested in the interviews.

#### Management of febrile patients

The survey showed that management of febrile patients was an important strategy for COVID-19 prevention and control. To respond to infectious diseases, in China, the corresponding departments have been established in the secondary hospitals and above, since the outbreak of SARS in 2003. The requirements have been standardized for setting up fever clinics, and for centralizing management of febrile patients to prevent and control infectious diseases [29]. However, team members from Zunyi reported the gap in febrile patient management. They found it was inconvenient for fever patients from remote regions, especially for those have not been exposed to any risk factor of COVID infection in asking for medical help. All febrile patients here should be transferred to the fever clinic of the designed secondary hospital during the pre-outbreak and outbreak period. Things was changed after fever sentinel clinic settings at PHC facilities.

#### Transferring

Our investigation showed that transferring was an important task for the PHC facilities in COVID-19 prevention and control. Besides ensuring patient safety, much attention is desired during transferring to protect the personnel and block all routes from causing the spread of the virus. In Shanghai and Zunyi, the transferring was performed on ambulances. Protected transferring by emergency medical service were also proved as one of the effective ways for COVID-19 containment in Israel [30]. With a high demand for transferring in the high-risk city of Wuhan, the provision of ambulances was not sufficient enough to transfer all of those in need, so the committee-owned vehicles became an effective alternative way. The recommendation is that transferring should be arranged by the government or by the health service providers in the prevention and control of infectious disease, and that the staff members should be well trained so that the risk of virus dissemination can be minimized.

### Task Difference in other countries

During the period of regular prevention and control of COVID-19 pandemic, confirmed COVID-19 patients’ treatment were managed by specialized teams at designed hospitals here in China. Their close contacts and returnees from other high-risk countries were individuals at-high risk of infection and they were managed at centralized quarantine centers. The preventing and controlling methods were different in other countries where the COVID-19 patients could be monitored by at-home quarantine with symptoms surveillance and by throat swabs detection [31, 32].

In China, only the special populations (Fig. 1) with negative NAT results and with proper housing conditions can apply for at-home quarantine and health observation after strict evaluation and approval. The main aim of quarantine management is to monitor patients and limit the transmission risk. The decision on quarantine at-home quarantine or centralized quarantine centers could be made by the estimation of specific pandemic situations in different regions.

The proper reconstruction of general hospitals to designated hospitals and hotels to quarantine centers were significantly crucial in preventing COVID-19 nosocomial infection. In Bangladesh, the lack of well preparation of facilities to certain hospitals during COVID-19 patients’ treatment in the early stages caused a high number of medical staffs infected with SARS-CoV-2 [33]. However, according to PCP 23, after carefully preparation, a maternity hospital was reconstructed to be a designated hospital and all medical and non-medical members didn’t get infected with SARS-CoV-2 in Wuhan.

### Implications for policy, practice and research

The PHC facilities in China were competent in both the surveillance and prevention and control tasks during COVID-19 pandemic. Emergency response was immediately needed once an emerging respiratory infectious disease was identified, so essential medical supply reserves should be guaranteed. Temporarily converted quarantine wards and centralized quarantine centers were successful attempts in managing sharply increased patients and populations at high-risk during a critically short time. Fever sentinel clinics at PHC facilities were important for disease surveillance, diagnosis and treatment of febrile patients, and competency improving of primary care systems in China. Moreover, psychological intervention for publics and medical staffs should not be overlooked.

## Conclusions

The main tasks of PHC facilities in China including assisting in contact tracing and epidemiological investigation, screening of populations at high-risk, at-home/centralized quarantine management and fever sentinel clinics/fever clinics work. Essential medical supply reserves at PHC facilities in mainland China were recommended in case of necessity. In addition to the construction of shelter hospitals and infectious disease hospitals, temporarily converted quarantine wards and centralized quarantine centers could also be available for emergency utilization. At-home quarantine strategy should be used discreetly than centralized quarantine. Fever sentinel clinics at PHC facilities was important in disease surveillance and clinical treatment. Patient explanations or psychological counseling was sometimes in need and really helped. Our findings are highly beneficial for healthcare teams and governments in handling similar situations.

## Supplementary Information

**Additional file 1.**

## Data Availability

All data relevant to the study was available from the corresponding author on reasonable request.
