# Supplementary material for: Prevention and control of COVID-19 by primary health care facilities in China: a field-survey-based qualitative study in three typical cities
Source: BMC Health Serv Res. 2022 Mar 26;22:399. doi: 10.1186/s12913-022-07770-4 (PMC8960212; doi:10.1186/s12913-022-07770-4)
Supplement: Supplementary file 1 — Additional file 1. [file 12913_2022_7770_MOESM1_ESM.docx]

**Contents**

[Consent to participate 2](#_Toc89073040)

[Figures and Tables 2](#_Toc89073041)

[Figure. S1 Flowchart of ward reconstruction for quarantine 2](file:///C:\Users\Yun\Desktop\现场调查%20BMC%20HSR投稿修改后1127\Supplementary%20Materials1127.docx#_Toc89073042)

[Figure. S2 Flowchart of tasks for at-home quarantine and observation 3](file:///C:\Users\Yun\Desktop\现场调查%20BMC%20HSR投稿修改后1127\Supplementary%20Materials1127.docx#_Toc89073043)

[Figure. S3 Flowchart of tasks in centralized quarantine sites 4](file:///C:\Users\Yun\Desktop\现场调查%20BMC%20HSR投稿修改后1127\Supplementary%20Materials1127.docx#_Toc89073044)

[Figure. S4 Flowchart of screening and management 5](file:///C:\Users\Yun\Desktop\现场调查%20BMC%20HSR投稿修改后1127\Supplementary%20Materials1127.docx#_Toc89073045)

[Table S1 Basic information of 22 PHC Facilities under field survey 6](#_Toc89073046)

[Table S2 Tasks of the PHC facilities during the pre-outbreak period 7](#_Toc89073047)

[Table S3 Tasks of COVID-19 prevention and control by the PHC facilities in cities at different risk levels during pre-outbreak period 7](#_Toc89073048)

[Table S4 Tasks of the PHC facilities during the regular prevention and control period 9](#_Toc89073049)

# Consent to participate

Verbal informed consents were obtained from of the medical workers and residents for participation in this study. The study protocol was approved by the Ethics Committee of Pudong Institute for Health Development, Shanghai.

# Figures and Tables

Disinfection of the entire hospital, planning three zones and two passages, planning quarantine wards, and one person/one ward management

Training on self-protection for all staff: hand washing, use of protective devices, dressing and undressing of protective suit, aseptic technique

Protective and medical supplies

All staff to live in a hotel, temporary canteen reconstruction

Medical staff: doctors and nurses from the PHC facilities, the Red Cross and those specialized in dentistry and obstetrics and gynecology; Non-medical staff: logistics personnel and volunteers

Preparation of housing environments

Staff

Preparation of medical supplies

Staff training on self-protection

Other supports

Ward reconstruction for quarantine

## Figure. S1 Flowchart of ward reconstruction for quarantine

This flowchart was concluded from expert interviews at the PHC facilities in Wuhan. Wuhan Red Cross Hospital as the example: The maternity hospital repurposed to be a designated hospital with 230 beds (one person/one ward); staff working on 24-hour shifts for more than 3 months, none of them infected. The ward reconstruction for quarantine was not performed at the PHC facilities in Shanghai and Zunyi, respectively

Visits by a three-person team composed of a neighborhood committee member, a public security officer and a doctor

The local government receiving the list

The list issued to the PHC facilities

Public security officer to install the “door magnet” and provide instructions

The first visit by medical staff:

-To verify information, fill in notification sheet and basic information sheet;

-To distribute health education leaflets and complete body temperature record sheet;

-To distribute garbage bags, disinfectant tablets, face masks, thermometers;

-To provide guidance on disinfection and ventilation

Neighborhood committee: coordinate and guide the disposal of garbage and waste

Follow-up management:

-Home visits for temperature measurement (over 4 hrs between two measurements);

-Symptom monitoring;

-Sampling for NAT

Individuals testing negative at the end of quarantine to be released

Individuals testing positive or having a need for other medical treatment to be transferred to the designated hospital

## Figure. S2 Flowchart of tasks for at-home quarantine and observation

PHC =primary health care, NAT=nucleic acid testing.

At-home quarantine and observation managed at the PHC facilities in the cities at different risk levels; this flowchart concluded from the working experiences of the PHC facilities in Shanghai; at-home quarantine and observation becoming more refined in Shanghai under the great devotion of manpower, material and financial resources

## Figure. S3 Flowchart of tasks in centralized quarantine sites

Centralized quarantine and observation performed at the PHC facilities in the cities at different risk levels

Information registration, entry, medical record

Diagnosis and treatment

Disinfection

Daily life support

Environmental/

surface disinfection

Medical waste disposal, sewage treatment, disposal of daily supplies (such as bedding) after discharge

Waste disposal

Symptomatic treatment: oxygen therapy, antipyretics, traditional Chinese medicine; diagnosis and timely referral

Food delivery, etc.

Patients with suspected infection, close contacts, febrile patients

Centralized quarantine/observation sites

Screening at traffic checkpoints (highway exits, high-speed railway stations, airports, etc.)

Household screening by neighborhood committee staff and village doctors

Pre-examination and triage at the PHC facilities

Febrile patients

Fever clinics at the designated hospitals

Transferred by the ambulance or neighborhood committee staff

People traveling from high-risk areas

Centralized quarantine for 14 days

Negative in two results of NAT*

At-home quarantine for 7 days

The third time of NAT*

Negative and released from quarantine

Positive

Transferred to designated hospital by ambulance

## Figure. S4 Flowchart of screening and management

NAT=nucleic acid testing

Screening and management performed at the PHC facilities in the cities at different risk levels; this flowchart concluded from the expert interviews at the PHC facilities in Zunyi.

*Different to Zunyi; NAT performed at the intervals of 0 and 12^th^ day during 14 days’ quarantine in Wuhan; NAT performed at the intervals of 0, 3^rd^ and 12^th^ day during 14 days’ quarantine in Shanghai

## Table S1 Basic information of 22 PHC Facilities under field survey

| **City** | **Areas** | **Facilities** | **Time** |
| --- | --- | --- | --- |
| **Shanghai** | Urban areas | Dapuqiao Community Healthcare Center, Huangpu District | Nov.6, 2020 |
| ⋅⋅ | ⋅⋅ | Pengpu Community Healthcare Center, Jingan District | Nov.11, 2020 |
| ⋅⋅ | Urban–rural areas | Shanggang Community Healthcare Center, Pudong New District | Nov.2, 2020 |
|  | ⋅⋅ | Hongqiao Community Healthcare Center, Changning District | Nov.5, 2020 |
| ⋅⋅ | ⋅⋅ | Jiading Community Healthcare Center, Jiading District | Nov.16, 2020 |
| ⋅⋅ | ⋅⋅ | Zhangjiang Community Healthcare Center, Pudong New District | Nov.3, 2020 |
| ⋅⋅ | Rural areas | Datuan Community Healthcare Center, Pudong New District | Nov.17, 2020 |
| **Wuhan** | Urban areas | Shouyilu Street Community Healthcare Center, Wuchang District | Dec.8, 2020 |
| ⋅⋅ | ⋅⋅ | Huanghelou Street Community Healthcare Center, Wuchang District | Dec.10, 2020 |
| ⋅⋅ | ⋅⋅ | Xinhua Street Community Healthcare Center, Jianghan District | Dec.14, 2020 |
| ⋅⋅ | ⋅⋅ | Wuhan University of Technology Community Healthcare Center | Dec.15, 2020 |
| ⋅⋅ | Urban–rural areas | Baiyushan Street Community Healthcare Center, Qingshan District | Dec.14, 2020 |
| ⋅⋅ | ⋅⋅ | Xinmin Community Healthcare Center, Dunyang Street, Economic-Technological Development Area | Dec.10, 2020 |
| ⋅⋅ | ⋅⋅ | Zuoling Street Community Healthcare Center, Donghu High and New Technical Development Zone | Dec.8, 2020 |
| ⋅⋅ | Rural areas | Sanliqiao Township health center, Huangpi District | Dec.9, 2020 |
| **Ezhou*** | Urban areas | Yitingming Community Healthcare Center, Gulou Street | Dec.11, 2020 |
| **Zunyi** | Urban areas | Dalianban Community Healthcare Center, Huichuan District | Dec.18, 2020 |
| ⋅⋅ | ⋅⋅ | Zhongshan Community Healthcare Center, Honghuagang District | Dec.21, 2020 |
| ⋅⋅ | Urban–rural areas | Xiangkou Township health center, Honghuagang District | Dec.24, 2020 |
| ⋅⋅ | ⋅⋅ | Liyi Community Healthcare Center, Xinpu New Area | Dec.23, 2020 |
| ⋅⋅ | Rural areas | Xinzhou Township Central health center, Honghuagang District | Dec.23, 2020 |
| ⋅⋅ | ⋅⋅ | Yachuan Township health center, Fenggang County | Dec.21, 2020 |

***** A city adjacent to Wuhan

## Table S2 Tasks of the PHC facilities during the pre-outbreak period

| **Tasks** |
| --- |
| **1. Storing supplies**  **1.1. Storing personal protective equipment:** face masks, gloves, protective suits  **1.2 Storing temporary substitutes for the protective materials in short supply:** raincoats and shoe covers  **1.3 Storing drugs:** Chinese patent medicine of Lianhua Qingwen, antiviral medication of ostavis and disinfectants |
| **2. Establishing working systems**  **2.1 Emergency working plans and drills**  **2.2 Personnel training coverage:** personal protective materials, clinical characteristics of COVID-19, diagnosis and treatment protocols and health education |
| **3. Others:** Strengthening prevention and control of hospital-acquired infections, strictly conducting pre-examination and triage, strengthening/improving fever (sentinel) consultation rooms/clinics, and reinforcing self-protection measures |

## Table S3 Tasks of COVID-19 prevention and control by the PHC facilities in cities at different risk levels during the outbreak period

| **Cities/tasks** |
| --- |
| **1 Wuhan as high-risk city** |
| **1. 1 Controlling infection sources** |
| **1) Screening:** assisting CDC in contact tracing and epidemiological investigation, screening at travel centers/terminals, pre-examination/triage, fever consultation rooms/clinics, collection of specimens from employees and items at cold-chain/fresh markets for NAT, collection of specimens from all residents for NAT*  **2) Transferring:** critically ill patients*, patients with suspected infection*, close contacts, febrile patients  **3) Quarantine:** at-home/centralized quarantine and observation  **4) Treatment*:** confirmed patients with mild symptoms, suspected patients |
| - 1. **Cutting off transmission routes** |
| 1) Household disinfection for confirmed patients  2) Temporary storage and disposal of waste  3) Environment disinfection  4) Handling dead bodies |
| **1.3 Protecting susceptible individuals** |
| 1) Face mask, hand washing, ventilation, social distancing  2) Health education  3) Medical support for those who were retained for settlement in Wuhan *  4) Health management of discharged patients  5) Support for resumption of work, production, and school classes  6) Psychological interventions  7) Vaccination against COVID-19 infection |
| **2 Shanghai as medium-risk city** |
| **2. 1 Controlling infection sources** |
| **1) Screening:** assisting CDC in contact tracing and epidemiological investigation, screening at travel centers/terminals, screening people traveling from high-risk areas using big data*, pre-examination/triage, fever (sentinel) consultation rooms/clinics, collection of specimens from employees and items at cold-chain/fresh markets for NAT  **2) Transferring:** febrile patients, people traveling from high-risk areas*  **3) Quarantine:** at-home/centralized quarantine and observation |
| **2.2 Cutting off transmission routes** |
| 1) Household disinfection for confirmed patients  2) Temporary storage and disposal of waste  3) Environment disinfection |
| **2.3 Protecting susceptible populations** |
| 1) Face mask, hand washing, ventilation, social distancing  2) Health education  3) Household health management of discharged confirmed/suspected patients  4) Supportive of the resumption of work, production and school classes  5) Psychological interventions  6) Vaccination against COVID-19 infection |
| **3 Zunyi as low-risk city** |
| **3.1 Controlling infection sources** |
| **1) Screening:** assisting CDC in contact tracing and epidemiological investigation; screening at travel centers/terminals, screening travelers from high-risk areas house-by-house*; performing pre-examination/triage in fever clinics; collecting NAT specimens from the employees and items at cold-chain/fresh markets  **2) Transferring:** febrile patients, travelers from high-risk areas*  **3) Quarantine:** at-home/centralized quarantine and observation |
| **3.2 Cutting off transmission routes** |
| **1)** Temporary storage and disposal of waste  **2)** Environment disinfection |
| **3.3 Protecting susceptible populations** |
| **1)** Face mask wearing, hand washing, ventilation, social distancing  **2)** Health education  **3)** Supportive of the resumption of work, production and school classes  **4)** Psychological interventions  **5)** Vaccination against COVID-19 infection |

*Typical tasks at the PHC facilities in the cities at different risk levels

CDC=Center for Disease Control and Prevention, NAT=Nucleic Acid Testing

The main tasks at the PHC facilities in Wuhan included treatment for confirmed and suspected patients; the main tasks at the PHC facilities in Zunyi were fundamental

## Table S4 Tasks of the PHC facilities during the regular prevention and control period

| **Tasks** | **Contents** |
| --- | --- |
| Controlling infection sources | 1. Screening: Performed at the travel centers/intervals on the travelers from the medium- and high-risk areas/febrile patients, conducting pre-examination and triage for febrile patients, sampling for SARS-CoV-2 NAT of patients, cold chain/fresh food markets and logistics  2.Transferring: Performed by the grid managers of community, neighborhood or village committees using ambulances  3. Quarantine: centralized/home quarantine |
| Cutting off transmission routes | 1. Disinfection: Environmental disinfection and disinfection of living places and surroundings exposed to confirmed and suspected patients  2. Waste disposal: Disposing used face masks, medical waste and others exposed to confirmed/suspected patients |
| Protecting susceptible populations | 1. Health education on protective measures  2. Medical support for resumption of work, production, school classes and large events  3. Vaccination against COVID-19 infection |
| Management and control within the PHC facilities | 1. Supplies storage, disinfection, waste disposal  2. Equipment required in fever (sentinel) consultation rooms/clinics (e.g., facilities should be equipped with CT if possible)  3. Management and control measures for hospital-acquired infections: Proper and prompt hand disinfection, marking out of 1-meter-distance lines, interval seating, appointments for treatment, patient relocation, and attendant number limitation  4. Personnel training/self-protection  5. Emergency working system: Assigning work groups on epidemiological investigation, contact tracing, storing supplies, medical treatment, information reporting, health education, NAT sampling, environmental disinfection, emergency plans and drills, respectively  6. Information reporting |

PHC=Primary Health Care, NAT=Nucleic Acid Testing
